# Association of Interleukin 1 Receptor Antagonist (*IL1RN*) rs4251961 with Periodontitis Risk in Portuguese Postpartum Women: A Pilot Study

**DOI:** 10.3390/ijms27167310

**Published:** 2026-08-16

**Authors:** Joana Couceiro, Carlos Família, José Brito, José João Mendes, Pedro V. Baptista, Alexandra R. Fernandes, Alexandre Quintas

**Affiliations:** 1 UCIBIO—Applied Molecular Biosciences Unit, Department of Life Sciences, NOVA School of Science and Technology, NOVA University Lisbon, 2829-516 Caparica, Portugal; pmvb@fct.unl.pt (P.V.B.); ma.fernandes@fct.unl.pt (A.R.F.); 2 Associate Laboratory i4HB—Institute for Health and Bioeconomy, NOVA School of Science and Technology, NOVA University Lisbon, 2829-516 Caparica, Portugal; 3 Egas Moniz Center for Interdisciplinary Research (CiiEM), Egas Moniz School of Health & Science, 2829-511 Almada, Portugal; carlosfamilia@egasmoniz.edu.pt (C.F.); jbrito@egasmoniz.edu.pt (J.B.); jmendes@egasmoniz.edu.pt (J.J.M.); aquintas@egasmoniz.edu.pt (A.Q.); 4 Forensic and Psychology Sciences Laboratory Egas Moniz, Campus Universitário Quinta da Granja, 2829-511 Caparica, Portugal

**Keywords:** periodontitis, predisposition, *TNFA* rs1800629, *IL1RN* rs4251961

## Abstract

Periodontitis (PD), the second most prevalent oral disease, contributes to systemic health issues and results from a complex interaction between microbial dysbiosis and host immune responses, strongly shaped by genetic background. This pilot case–control study evaluated associations between five inflammation-related variants (*IL1A* rs1800587, *IL1B* rs1143634, *IL1B* rs16944, *IL1RN* rs4251961, *TNFA* rs1800629) and PD susceptibility in Portuguese postpartum women. Eighty-eight women (33 PD patients and 55 controls) were genotyped using iPLEX Gold technology. Associations were tested using logistic regression models, with multiple-testing correction applied to adjust significance thresholds. *TNFA* rs1800629 was associated with PD in the collapsed model (GA+AA vs. GG; OR = 3.3, *p* < 0.05). *IL1RN* rs4251961 showed strong associations across all models. In the codominant model, heterozygotes had increased PD odds (OR = 3.52, adjusted *p* < 0.05), while CC homozygotes showed 8-fold odds (OR = 8.1, adjusted *p* < 0.05). The collapsed model was also significant (*p* < 0.05). The remaining variants were not associated with PD. *IL1RN* rs4251961 may represent a novel genetic marker for PD, with clinical relevance given its inflammatory role and therapeutic use of recombinant IL-1RA in chronic inflammatory diseases. This first report linking *IL1RN* rs4251961 to PD supports validation in larger European cohorts.

## 1. Introduction

According to the latest World Health Organization (WHO) global health report, oral diseases represent a significant public health issue for countries and populations worldwide despite often not being publicly recognized. The global number of oral disease cases is estimated to be 1 billion higher than the combined number of cases across all five main noncommunicable diseases [1]. Periodontitis (PD) is the second most prevalent oral disease, with over 1 billion cases reported in 2019. The estimated global prevalence of severe periodontitis in adults and elderly individuals is at least 16.9% [1], and the 2019 Global Burden of Disease study revealed that oral diseases, including severe PD, cause 23.1 million disability-adjusted life-years worldwide [2]. Moreover, epidemiological, clinical interventional and experimental studies have demonstrated that PD adversely impacts systemic health, connecting this disease to several chronic conditions, such as type 2 diabetes, cardiovascular disease, Alzheimer’s disease, and certain cancers, among others [3]. PD is a chronic inflammatory disease that affects the integrity of the tissues supporting the teeth and results from a complex interplay between subgingival biofilm dysbiosis and the host immune response [4]. Although PD is influenced by multiple environmental and behavioural risk factors, considerable interindividual variability in disease progression suggests that there is a genetic component. Genetic factors are estimated to account for up to one-third of PD variance, with an even greater contribution in more severe forms of the disease [5]. Among the genes already suggested to be associated with PD, those related to inflammatory pathways are predominant; however, the results of the studies are inconsistent. Interleukin-1 (IL-1) and tumour necrosis factor (TNF-α) are the most studied inflammatory cytokine families in PD [6,7]. Both are pro-inflammatory cytokines associated with the pathogenesis of PD [8]. TNF-α is ubiquitously present in inflammatory responses and plays a critical role in the expression of many chemokines and other cytokines [9]. IL-1 alpha (IL-1A), IL-1 beta (IL-1B), and IL-1 receptor antagonist (IL-1RA), encoded by the *IL1RN* gene, are members of the IL-1 family of ligands, produced and secreted by many cells, and play crucial roles in activating and controlling inflammatory responses [10]. IL-1A and IL-1B are pro-inflammatory cytokines, while IL-1RA is the principal endogenous IL-1 antagonist, competitively binding to IL-1 receptors without inducing intracellular signalling, preventing IL-1A and IL-1B activity, and thereby acting as an inflammatory control protein. The pro-inflammatory response is initiated and persists or ends depending on the relative levels of IL-1RA and IL-1. Higher levels of IL-1RA reduce the severity of the inflammation [11]. Supporting these findings, IL-1RA knockout mice reveal excessive inflammatory responses and develop different spontaneous inflammatory diseases [12]. Finally, the clinical relevance of IL-1RA has also been demonstrated by the FDA approval of Anakinra, a recombinant human IL-1RA, for the treatment of rheumatoid arthritis and systemic juvenile idiopathic arthritis [13].

The most-studied polymorphisms in cytokine-encoding genes associated with PD are *IL1A* rs1800587 (−899), *IL1B* rs1143634 (+3953/4), *IL1B* rs16944 (−511), and *TNFA* rs1800629 (−308). However, the results of the different studies are inconsistent. Several candidate gene studies associated the *IL1A* rs1800587 with PD [14,15,16], while others reported no association [17,18,19]. Both *IL1B* variants, rs1143634 and rs16944, showed the same trend: some studies reported a correlation with PD, while others failed to demonstrate a significant association [16,18,19,20,21]. As with *IL1A* and *IL1B*, case–control studies examining the association between *TNFA* rs1800629 and PD susceptibility have reported inconsistent findings [22,23]. Contradictory results have been attributed to differences in the applied diagnostic criteria, the geographic distribution of specific gene variants [24,25], and sex dimorphism in PD, which is not entirely understood but may be related to differences in immune response between men and women [26].

Although IL-1RA plays a crucial role in providing protection against periodontal tissue destruction [6], only a limited number of *IL1RN* variants have been investigated in PD. For example, the *IL1RN* rs419598 polymorphism has been associated with reduced susceptibility to generalized PD in individuals of European ancestry [27]. However, *IL1RN* rs4251961 has not previously been investigated in this context. The minor C allele of *IL1RN* rs4251961 has been associated with reduced IL-1RA expression, leading to enhanced IL-1-mediated pro-inflammatory responses [12,13]. Therefore, *IL1RN* rs4251961 represents a plausible candidate variant for investigation with regard to PD susceptibility.

The present pilot study aimed to identify possible associations between *IL1A* rs1800587, *IL1B* rs1143634, *IL1B* rs16944, *IL1RN* rs4251961, and *TNFA* rs1800629 and the risk of PD. It addresses previously reported issues by narrowing down origin and sex dimorphism. Thus, this study was conducted in a cohort of Portuguese postpartum women.

## 2. Results

### 2.1. Demographic and Lifestyle Information of the Control Group and PD Group

Table 1 presents demographic and lifestyle data for patients with periodontitis and healthy subjects. The study included 55 controls, aged 31.9 ± 5.97 years, and 33 patients with PD, aged 30.2 ± 5.8 years. There were no significant differences in the age parameter between the two groups (*p* > 0.05). In addition, the number of women who smoked in the control and PD groups was six (10.9%) and five (15.2%), respectively. There were also no significant differences in smoking status between the two groups. Regarding ancestry, most subjects identified as European in both groups: 83.6% in the control group and 87.9% in the PD group. No significant differences were found in any demographic or lifestyle parameters, namely, level of education, occupation status, marital status, daily frequency of toothbrushing, or use of dental floss.

### 2.2. Genotype Frequencies in PD Patients and Healthy Controls

DNA from all the subjects was genotyped for five selected SNPs (*IL1A* rs1800587, *IL1B* rs1143634, *IL1B* rs16944, *IL1RN* rs4251961, and *TNFA* rs1800629) to evaluate their putative associations with PD risk. The five SNPs in the control group fulfilled the HWE criteria (*p* > 0.05). The genotype distributions for the five SNPs between cases and controls are shown in Table 2.

Table 3 shows the significances and their adjustments of the results in each of the models applied to assess the global effect of the genetic predictors. Significant associations for specific genetic variants were detected under the logistic regression framework, particularly for *IL1RN* rs4251961, and more moderately for *TNFA* rs1800629. The genotype frequencies of the two significant SNPs in the PD patients and healthy controls are presented in Table 4, which also shows the results of the Wald test with regard to the odds ratios (OR) in the corresponding model.

**Figure 1:**
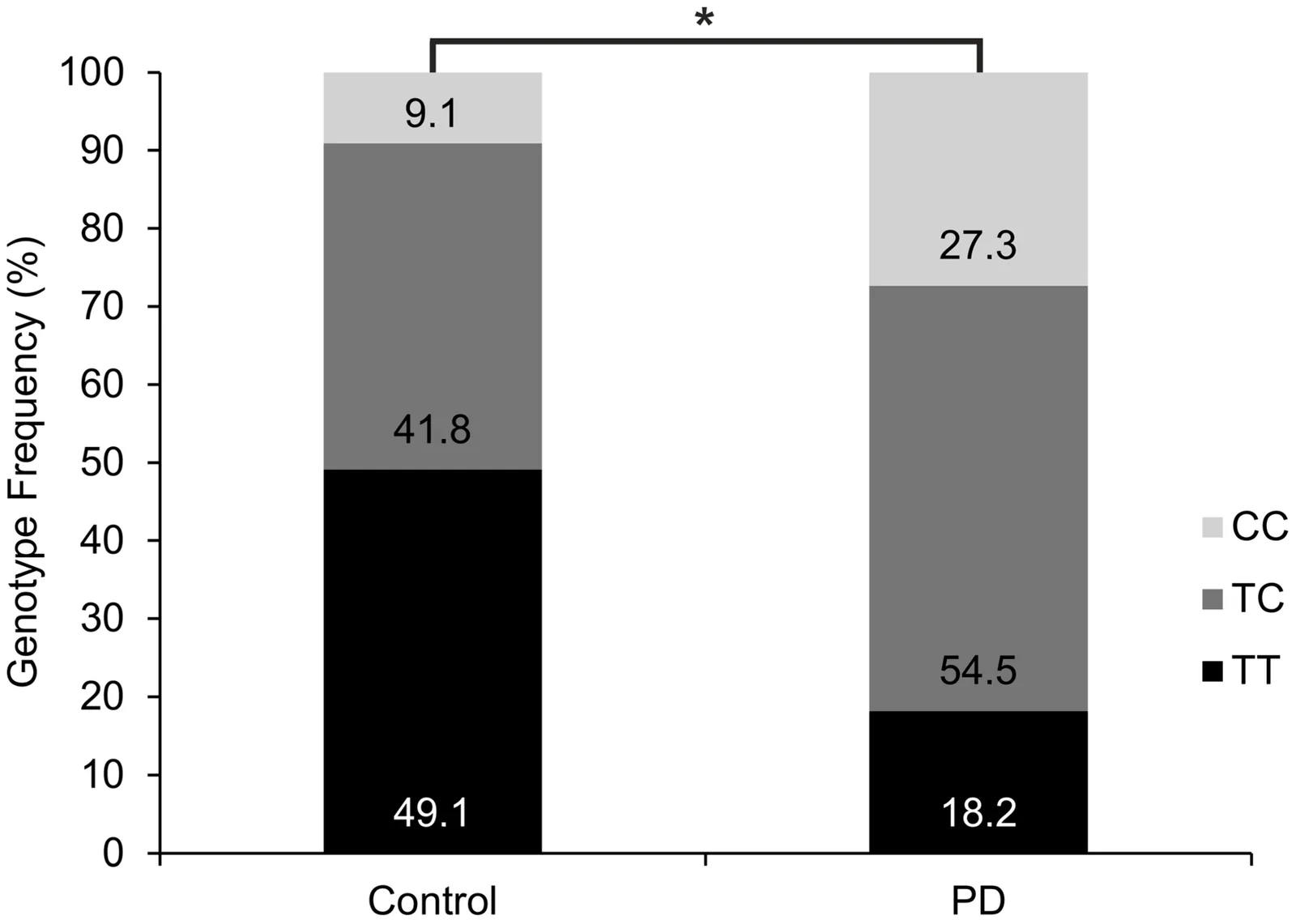
Visual representation of *IL1RN* rs4251961 genotype distributions in healthy controls and patients with PD. * Adjusted *p*-value ≤ 0.05.

The *IL1RN* rs4251961 is a strongly significant predictor for the PD, as the significance of both the full and the collapsed models for this SNP is demonstrated by adjusted *p*-values below 0.05 under BH and Bonferroni corrections. The adjusted *p*-value above 0.05 but below 0.1 under BY corrections also suggested the effect of the predictor (Table 3). As shown in Table 4, the *IL1RN*/TC genotype was associated with significantly higher odds of PD (OR = 3.52, 95% CI 1.20–10.35, all adjusted *p*-values < 0.05), compared with reference genotype TT. The *IL1RN*/CC genotype showed 8.10 times higher odds of PD (95% CI 1.98–33.05, all adjusted *p*-values < 0.05). Additionally, the collapsed model (TC+CC vs. TT) showed 4.34 times higher odds of PD (95% CI 1.55–12.16, *p*-value < 0.05). The distribution of *IL1RN* rs4251961 genotypes in controls and PD patients is depicted in Figure 1 to illustrate the significant differences in genotype frequencies between cases and controls. The *TNFA* rs1800629 showed a significant association with PD in the “collapse” model (GA+AA vs. GG) under BH correction, but not under Bonferroni or BY corrections, suggesting a potential association with PD that warrants further exploration. Nevertheless, the results suggest 3.32 higher odds of PD for the collapsed model, compared with the reference genotype *TNFA*/GG (OR = 3.2, 95% CI 1.25–8.77, BH adjusted *p*-value < 0.05).

As expected for a pilot study, despite the significant associations, the observed statistical power was lower than the desired 80% (Table 4). Based on these results, the estimated sample size required to strengthen the robustness of these results is 161 subjects.

## 3. Discussion

PD is a complex chronic inflammatory disease influenced by interactions between the host immune response, microbial dysbiosis, and the individual’s genetic background [4,5,28]. Although several cytokine-related genes have been associated with PD susceptibility, findings remain inconsistent, partly because of population-specific genetic variation and sex dimorphism [24,25,26]. Accordingly, in the present pilot study, we investigated the potential associations of *IL1A* rs1800587, *IL1B* rs1143634 and rs16944, *TNFA* rs1800629, and *IL1RN* rs4251961 with PD in a cohort of Portuguese women.

The main findings of the present study were the associations between *TNFA* rs1800629 and *IL1RN* rs4251961 variants and PD. *TNFA* rs1800629 showed a moderately significant association with PD, while *IL1RN* rs4251961 revealed a strongly significant association with the disease in both the full and collapsed models, even after BH and Bonferroni corrections. It is noteworthy to mention that this is the first study to investigate the *IL1RN* rs4251961 variant in the PD context.

TNF-α is one of the earliest and most important mediators of the inflammatory response and a pro-inflammatory cytokine involved in bone turnover. Higher levels of TNF-α increase osteoclast formation, which is responsible for dissolving old and damaged bone tissue [29]. Thus, genetic variants in its encoding gene that lead to overexpression or produce a higher-activity protein contribute to bone loss. The genetic variant analyzed in this study (rs1800629/−308) is a promotor polymorphism linked to enhanced *TNFA* expression [30,31], potentially contributing to the propensity for PD. The most recent meta-analysis of 24 studies, including PD cases with and without type 2 diabetes mellitus, also showed a positive association between this *TNFA* variant and PD in the collapsed model (AA+GA vs. GG) in overall analyses (OR = 1.46, 95% CI 1.12–1.89). However, after stratification by ancestry, the association between *TNFA* rs1800629 and PD was not significant in European populations across all tested models [32]. Interestingly, Li and colleagues (2020), in a meta-analysis of 36 studies, reported that most of the eligible studies were conducted among Asian populations, and there is a significant lack of research on other groups, suggesting that future genetic association studies should also explore the relationship between *TNFA* variants and PD in other populations [33]. *IL1RN* rs4251961 has never been studied previously in the context of PD; however, the correlation found was not surprising due to IL-1RA’s role in inflammation [12,13] and bone remodelling [34]. As IL-1 stimulates osteoblastic activity and osteoclastic differentiation [35], decreased IL-1RA function leads to higher levels of IL-1B, increasing the risk of bone loss [36], which in turn heightens susceptibility to PD [37] and marginal bone loss around dental implants [38]. In addition, the most studied variant of *IL1RN*, an 86 bp VNTR (rs2234663), which decreases IL-1RA expression, has been linked to several inflammatory and autoimmune diseases, including ulcerative colitis [39], systemic lupus erythematosus [40], and even PD [19,20,41]. Remarkably, the *IL1RN* variant investigated in this pilot study, rs4251961, is an SNP associated with lower serum IL-1RA concentrations than those previously reported for the *IL1RN* VNTR [41,42]. Although *IL1RN* genetic variants were not fully explored in PD, it is well known that IL-1RA plays a defence role in the disease, and its deficiency contributes to severe periodontal tissue destruction [6,43].

*TNFA* and *IL1RN* genetic variants exhibit significant variability across different ancestries and between sexes, influencing health outcomes and inflammatory response [19,20,44]. Our pilot study addressed prior concerns about the frequency of genetic variants across ancestries, explicitly noting the lack of research on the *TNFA* rs1800629 variant in European-descent populations. Additionally, we considered sex differences, focusing exclusively on women. Our findings reveal a significant association between the *IL1RN* rs4251961 variant and PD within the logistic regression framework. Notably, despite applying stringent significance corrections with the Benjamini–Hochberg and Bonferroni methods, the effect of this genetic variant on disease susceptibility remained robust. These findings underscore the importance of this polymorphism as a potential genetic marker for the disease. Moreover, moderately significant results for *TNFA* rs1800629 also suggest its relevance for PD. The three other variants studied, *IL1A* rs1800587, *IL1B* rs1143634, and rs16944, were not significant predictors. All failed to achieve significance after adjustment, reflecting weaker associations or insufficient evidence of an effect on PD.

As expected, the main limitation of our pilot study is the sample size (N = 88), which may be insufficient for detecting moderate effect sizes, especially given the binary nature of the outcome. The power was further reduced by multiple testing adjustments, with Bonferroni, Benjamini–Hochberg, and Benjamini–Yekutieli corrections lowering the significance threshold, increasing the likelihood of Type II error. Additionally, the effect size, while notable (e.g., OR = 3.52 for the *IL1RN*/TC genotype), is not large enough to compensate for sample-size constraints, particularly if the distribution of subjects across genotype categories is unbalanced. The wide confidence intervals, especially for the *IL1RN*/CC genotype, further suggest instability in the estimates due to limited event counts. Another aspect to consider is the characteristics of the study population. Although participants were recruited during the postpartum period, pregnancy-associated hormonal and immunological changes are primarily linked to pregnancy-associated gingivitis, a transient and generally reversible condition, rather than PD [45]. Nevertheless, the present findings should be interpreted as genetic associations observed in this specific population. Further studies including women at other stages of life will be important to determine the generalizability of these findings. Overall, this pilot study provides preliminary evidence supporting an association between *IL1RN* rs4251961 and PD within this Portuguese postpartum population. However, future studies with larger sample sizes are needed to confirm and validate this association in individuals of European ancestry. To increase the statistical power of future studies on this subject, collaborative research protocols involving dental clinic networks or governmental primary health care networks should be encouraged. Regardless, the present study contributes to the understanding of the genetic background of PD susceptibility by highlighting *IL1RN* rs4251961 as a candidate variant for further investigation, while also emphasizing the importance of considering sex and ancestry in genetic association studies. In conclusion, our findings suggest that *IL1RN* rs4251961 may represent a candidate susceptibility marker for PD, warranting further validation in independent cohorts.

## 4. Materials and Methods

### 4.1. Study Population

The study population consisted of postpartum women followed at the Puerperium of the Service of Gynaecology and Obstetrics from Hospital Garcia de Orta (HGO), Portugal, recruited between 2 March 2020, and 5 May 2022. The study protocol was approved by the HGO ethics committee (56/2019). All the women provided fully informed written consent to participate and completed the questionnaire, which included questions on sociodemographic and lifestyle characteristics, such as level of education, occupation status, smoking habits, toothbrushing frequency, and flossing frequency. Clinical diagnosis and blood sample collection were carried out up to 72 h after delivery. All experimental methods throughout the study were conducted in accordance with the Declaration of Helsinki and the ethical principles for medical research involving human subjects.

As this was an exploratory pilot study, no formal a priori sample size calculation was performed. All eligible women recruited during the study period who completed the study assessments and fulfilled the inclusion criteria were included. The completed STROBE checklist is available in the Supplementary Materials.

### 4.2. Selection of Cases and Control Subjects

A full-mouth periodontal examination was performed for PD diagnosis. No radiographic examination was performed. All fully erupted teeth, except for the third molars, dental implants, and retained roots, were evaluated via a manual periodontal North Carolina probe (Hu-Friedy, Chicago, IL, USA). The number of missing teeth, plaque index, gingival recession (REC), periodontal probing depth (PPD), and bleeding on probing (BoP) were recorded at six sites per tooth (mesiobuccal, mid-buccal, distobuccal, mesiolingual/palatal, mid-lingual/palatal, and distolingual/palatal). PPD was measured as the distance from the free gingival margin to the bottom of the periodontal pocket, and REC was defined as the distance from the cementoenamel junction to the free gingival margin. The clinical attachment level was calculated as the algebraic sum of the REC and PPD measurements. Furcation involvement was assessed using a 2 N probe (Hu-Friedy; Chicago, IL, USA) in molars and upper first premolars. PD cases were diagnosed by experienced dentists from the Clinical Research Unit of Egas Moniz, following standardized clinical procedures according to the 2018 AAP/EFP classification criteria [46]. Following examination, each woman was informed of their periodontal status and, if diagnosed with periodontal disease (either gingivitis or periodontitis), was referred to the Egas Moniz Dental Clinic for treatment without cost. Among the 95 subjects, 33 women had PD. The remaining subjects, who did not meet the diagnostic criteria for PD, were eligible for use as healthy controls, but seven women were excluded due to missing data on the variables under study; thus, 55 women were enrolled as control subjects. Immediately after PD diagnostics, a 2 mL peripheral blood sample was collected in EDTA-coated tubes (BD Vacutainer^®^, Becton, Dickinson and Company, Franklin Lakes, NJ, USA) from all enrolled participants (patients and controls), who were fully anonymized using a code by hospital personnel, so that the molecular laboratory technician was blinded to patients’ information and group allocation. The samples were immediately transported under refrigeration to the molecular laboratory at Egas Moniz School of Health & Science or the NOVA FCT Lab and kept at 4 °C (for a maximum of 1 day) before undergoing further processing for molecular analysis.

### 4.3. DNA Extraction and Genotyping

DNA was extracted using the NZY Tissue gDNA Isolation Kit (NZYTech, Lisbon, Portugal) according to the manufacturer’s instructions and stored at −80 °C until use. Genotyping was carried out at CEGEN-FPGMX (Fundación Pública Galega de Medicina Xenómica, Santiago de Compostela, Spain) using the iPLEX Gold technology (Agena Bioscience, San Diego, CA, USA) as a service. Five single-nucleotide polymorphisms (SNPs) in *IL1A* (HGNC ID: 5991), *IL1B* (HGNC ID: 5992), *TNFA* (HGNC ID: 11892), and *IL1RN* (HGNC ID: 6000) genes were typed, namely, *IL1A* rs1800587 (−889), *IL1B* rs1143634 (+3954), *IL1B* rs16944 (−511), *IL1RN* rs4251961 (−1129), and *TNFA* rs1800629 (−308). Genotyping quality control was performed; the overall SNP call rate (genotyping success rate) across the five markers was 97.45%. Furthermore, the methodological rigour was confirmed by genotyping >20% of samples in duplicate, and the successful genotyping of an internal Coriell DNA familial trio used as a positive control, which showed a 100% call rate.

### 4.4. Statistical Analysis

Statistical analysis of the questionnaire data, including sample characterization, descriptive statistics and assessment of the differences between groups for sociodemographic and lifestyle characteristics (using chi-square/Fisher’s exact test or Student’s *t*-test), was performed using R version 4.2.1 (R Foundation for Statistical Computing, Vienna, Austria). The chi-square test was used to evaluate SNPs within the healthy control group for potential deviations from Hardy–Weinberg equilibrium. Additionally, logistic regression models were used to assess the role of genetic predictors in explaining the presence or absence of PD. Firstly, the likelihood ratio test was applied to estimate the global effect of the predictors on PD. Secondly, whenever such an effect was significant, the Wald test was applied to determine whether the odds ratios for each predictor category were significant relative to a reference category. Adjustments to the significance of the results in each model were implemented using multiple correction methods, to control the family-wise error rate (FWER) and the false-discovery rate (FDR) in view of the number of simultaneously tested hypotheses. A total of 10 logistic models were constructed, involving the five genetic variants under study: *IL1RN* rs4251961, *TNFA* rs1800629, *IL1A* rs1143634, *IL1B* rs16944, and *IL1B* rs1800587. For each gene, two models were considered, namely (i) the full models, with all three genotypic categories, and (ii) the “collapsed models,” in which the two possible genotypes with the minor allele were collapsed into one category, with the homozygote for the major allele being the alternative genotype (reference genotype). All procedures for the logistic regression analysis were performed using IBM SPSS Statistics version 29 (IBM Corp., Armonk, NY, USA).

Given the simultaneous testing of 10 models, three correction methods were applied to control the FWER and the FDR: (i) Bonferroni correction, to control FWER by dividing the significance level (α) by the number of tests; (ii) Benjamini–Hochberg (BH), to control FDR under the assumption of independent or positively correlated tests; and (iii) Benjamini–Yekutieli (BY), to control FDR under any dependency structure among tests. It is important to note that the BH correction is less conservative than Bonferroni, allowing for the identification of potentially significant effects while controlling the FDR, whereas BY correction is highly conservative, particularly when dependencies among tests are suspected, resulting in higher adjusted *p*-values. For the results interpretation, predictors were considered “strongly significant” if the significance was demonstrated by adjusted *p*-values below 0.05 under BH and Bonferroni corrections and between 0.05 and 0.1 under BY. Predictors were considered “moderately significant” if they showed significance after BH correction, but not under Bonferroni or BY corrections, suggesting a potential association with PD that warrants further exploration. Finally, predictors that failed to achieve significance after adjustment were considered “non-significant”. Observed power calculations and computations of required sample size to achieve the 80% power threshold were performed with the GPower version 3.1.9.6 software (Heinrich Heine University Dusseldorf, Dusseldorf, Germany).

## Figures and Tables

**Table 1:** Demographic and lifestyle characteristics of patients with PD and healthy controls.

	Control (N = 55)	PD (N = 33)	*p* Value
Age			
Mean (SD)	31.9 (5.97)	30.2 (5.80)	0.206
Median [Min, Max]	32.0 [18.0, 43.0]	30.0 [22.0, 43.0]	
Ancestry			
European	46 (83.6%)	29 (87.9%)	0.320
Education level			
Uneducated	1 (1.8%)	1 (3.0%)	0.807
Elementary school	7 (12.7%)	4 (12.1%)	
High school	25 (45.5%)	18 (54.5%)	
Graduate	22 (40.0%)	10 (30.3%)	
Occupation status			
Student	2 (3.6%)	1 (3.0%)	1.000
Active	40 (72.7%)	24 (72.7%)	
Unemployed	13 (23.6%)	8 (24.2%)	
Marital status			
Single	21 (38.2%)	16 (48.5%)	0.774
Cohabiting	14 (25.5%)	8 (24.2%)	
Married	17 (30.9%)	8 (24.2%)	
Divorced	3 (5.5%)	1 (3.0%)	
Smoker			
Yes	6 (10.9%)	5 (15.2%)	0.741
Daily tooth brushing frequency			
Once or less	5 (9.1%)	2 (6.1%)	0.725
Twice	37 (67.3%)	20 (60.6%)	
Three or more	13 (23.6%)	10 (30.3%)	
Missing	0 (0%)	1 (3.0%)	
Floss usage			
No	13 (23.6%)	12 (36.4%)	0.213
Occasionally	4 (7.3%)	4 (12.1%)	
One to three times a week	16 (29.1%)	4 (12.1%)	
Everyday	22 (40.0%)	12 (36.4%)	
Missing	0 (0%)	1 (3.0%)	

N—number of participants; PD—patients with periodontitis; SD—standard deviation.

**Table 2:** Distributions of the three genotypic categories of selected SNPs (*IL1A* rs1800587, *IL1B* rs1143634, *IL1B* rs16944, *IL1RN* rs4251961, and *TNFA* rs1800629).

SNP	Genotype	Control	PD
		N (%)	N (%)
*Il1RN* rs4251961	TT	27 (49.1)	6 (18.2)
	TC	23 (41.8)	18 (54.5)
	CC	5 (9.1)	9 (27.3)
*TNFA* rs1800629	GG	45 (81.8)	19 (57.6)
	GA	10 (18.2)	13 (39.4)
	AA	0 (0.0)	1 (3.0)
*IL1B* rs1143634	GG	32 (58.2)	17 (51.5)
	GA	18 (32.7)	16 (48.5)
	AA	5 (9.1)	0 (0.0)
*IL1B* rs16944	AA	6 (10.9)	7(21.2)
	AG	23 (41.8)	13 (39.4)
	GG	26 (47.3)	13 (39.4)
*IL1A* rs1800587	GG	20 (36.4)	14 (42.4)
	GA	28 (50.9)	17 (51.5)
	AA	7 (12.7)	2 (6.1)

N—number of participants; PD—patients with periodontitis.

**Table 3:** Results of likelihood tests of global effects of genetic predictors: ordered *p*-values and their adjustments for the 10 logistic models.

Model	*p* Value	BH Adjusted *p*	BY Adjusted *p*	B Adjusted *p*
rs4251961_coll	0.003	0.025 *	0.073	0.030 *
rs4251961	0.005	0.025 *	0.073	0.050 *
rs1800629_coll	0.014	0.047 *	0.137	0.140
rs1800629	0.029	0.073	0.212	0.290
rs1143634_coll	0.543	0.573	1.000	1.000
rs1143634	0.046	0.092	0.269	0.460
rs16944_coll	0.194	0.323	0.947	1.000
rs16944	0.417	0.573	1.000	1.000
rs1800587_coll	0.573	0.573	1.000	1.000
rs1800587	0.559	0.573	1.000	1.000

B—Bonferroni; BH—Benjamini–Hochberg; BY—Benjamini–Yekutieli corrections; coll—collapsed model; * Polymorphisms with statistically significant associations with PD (adjusted *p* < 0.05).

**Table 4:** Genotype distributions of *IL1RN* rs4251961 and *TNFA* rs1800629 in patients with PD and controls, significances of the Wald test results and power calculations.

SNP	Genotype	Control	PD	OR	95% CI	*p* Value	*p* Value ^a^	Observed Power	Sample Size 80% Power
		N (%)	N (%)						
*Il1RN* rs4251961	TT	27 (49.1)	6 (18.2)	1 (ref)					
	TC	23 (41.8)	18 (54.5)	3.52	1.20–10.35	0.022	0.022 (BH), 0.033 (BY), 0.044 (B)	0.493	154
	CC	5 (9.1)	9 (27.3)	8.10	1.98–33.05	0.004	0.008 (BH), 0.012 (BH), 0.008 (B)	0.758	97
	TC+CC	28	27	4.34	1.55–12.16	0.005	0.005 (NA)	0.520	138
*TNFA* rs1800629	GG	45 (81.8)	19 (57.6)	1 (ref)					
	GA+AA	10	14	3.32	1.25–8.77	0.016	0.016 (NA)	0.494	161

B—Bonferroni correction; BH—Benjamini–Hochberg correction; BY—Benjamini–Yekutieli correction; CI—confidence interval; N—number of alleles; NA—not applicable, as no multiple-testing adjustment was required for the collapsed model; OR—odds ratio; PD—patients with periodontitis; ref—reference genotype; SNP—single-nucleotide polymorphism; ^a^—adjusted *p*-value.

## Data Availability

The data presented in this study are not publicly available due to ethical and privacy restrictions related to the genetic and clinical information of the study participants. Access to anonymized data may be considered by the corresponding author upon reasonable request, subject to approval by the relevant ethics committee and institutional regulations.

## Supplementary Materials

The following supporting information can be downloaded at https://www.mdpi.com/article/10.3390/ijms27167310/s1.

## Abbreviations

The following abbreviations are used in this manuscript:

PD	Periodontitis
REC	Gingival recession
PPD	Periodontal probing depth
SNPs	Single-nucleotide polymorphisms
FWER	Family-wise error rate
FDR	False-discovery rate
HGO	Hospital Garcia de Orta
