# Supplementary material for: Association of Interleukin 1 Receptor Antagonist (IL1RN) rs4251961 with Periodontitis Risk in Portuguese Postpartum Women: A Pilot Study
Source: Int J Mol Sci. 2026 Aug 16;27(16):7310. doi: 10.3390/ijms27167310 (PMC13513210; doi:10.3390/ijms27167310)
Supplement: Supplementary file 1 [file ijms-27-07310-s001.zip › ijms-4460935-supplementary.pdf]

STROBE Statement—checklist of items that should be included in reports of observational studies

	Item No.	Recommendation	Page No.	Relevant text from manuscript
**Title and abstract**	1	(*a*) Indicate the study's design with a commonly used term in the title or the abstract	1	**Title:** "…: a pilot study"; **Abstract:** "This pilot case-control study…"
		(*b*) Provide in the abstract an informative and balanced summary of what was done and what was found	1	"Eighty-eight women (33 PD patients, 55 controls) were genotyped…" in methods section. "TNFA rs1800629 was associated with PD… IL1RN rs4251961 showed strong associations across all models…" in results section.
**Introduction**				
Background/rationale	2	Explain the scientific background and rationale for the investigation being reported	1-3	"…the results of the studies are inconsistent… Although IL-1RA plays a crucial role in protecting against periodontal tissue destruction, only a limited number of IL1RN variants have been investigated in PD… However, IL1RN rs4251961 has not previously been investigated in this context."
Objectives	3	State specific objectives, including any prespecified hypotheses	3	"The present pilot study aimed to identify possible associations between IL1A rs1800587, IL1B rs1143634, IL1B rs16944, IL1RN rs4251961, and TNFA rs1800629 and the risk of PD."

**Methods**				
Study design	4	Present key elements of study design early in the paper	3	"The present pilot study aimed to identify possible associations between IL1A rs1800587, IL1B rs1143634, IL1B rs16944, IL1RN rs4251961, and TNFA rs1800629 and the risk of PD…Thus, this study was conducted in a cohort of Portuguese postpartum women." in introduction section.
Setting	5	Describe the setting, locations, and relevant dates, including periods of recruitment, exposure, follow-up, and data collection	10	"The study population consisted of postpartum women followed at the Puerperium of the Service of Gynaecology and Obstetrics from Hospital Garcia de Orta (HGO), Portugal, recruited between March 2, 2020, and May 5, 2022" in methods section
Participants	6	(*a*) *Case-control study*—Give the eligibility criteria, and the sources and methods of case ascertainment and control selection. Give the rationale for the choice of cases and controls	10, 11	"The study population consisted of postpartum women… All the women provided fully informed written consent to participate and completed the questionnaire… A full-mouth periodontal examination was performed for PD diagnosis… PD cases were diagnosed by experienced dentists…according to the 2018 AAP/EFP classification criteria…The remaining subjects, who did not meet the diagnostic criteria for PD, were eligible for

				use as healthy controls, but seven women were excluded due to missing data on the variables under study”
		(*b*) *Case-control study*—For matched studies, give matching criteria and the number of controls per case	N/A	Not applicable (unmatched case-control study)
Variables	7	Clearly define all outcomes, exposures, predictors, potential confounders, and effect modifiers. Give diagnostic criteria, if applicable	10, 11	“…the questionnaire, which included questions on socio-demographic and lifestyle characteristics, such as level of education, occupation status, smoking habits, toothbrushing frequency, and flossing frequency… PD cases were diagnosed by experienced dentists… following standardized clinical procedures according to the 2018 AAP/EFP classification criteria… Five single nucleotide polymorphisms (SNPs) in IL1A (HGNC ID: 5991), IL1B (HGNC ID: 5992), TNFA (HGNC ID: 11892), and IL1RN (HGNC ID: 6000) genes were typed, namely, IL1A rs1800587 (-889), IL1B rs1143634 (+3954), IL1B rs16944 (-511), IL1RN rs4251961 (-1129), and TNFA rs1800629 (-308).”
Data sources/ measurement	8*	For each variable of interest, give sources of data and details of methods of assessment (measurement). Describe comparability of assessment methods if there is more than one group	10, 11	“All the women…completed the questionnaire… All fully erupted teeth, except for the third molars,

				dental implants, and retained roots, were evaluated via a manual periodontal North Carolina probe (Hu-Friedy, Chicago, Illinois, USA). The number of missing teeth, plaque index, gingival recession (REC), periodontal probing depth (PPD), and bleeding on probing (BoP) were recorded at six sites per tooth… PD cases were diagnosed by experienced dentists… following standardized clinical procedures according to the 2018 AAP/EFP classification criteria…a 2 mL peripheral blood sample was collected in EDTA-coated tubes from all enrolled participants (patients and controls), who were fully anonymized using a code by hospital personnel, so that the molecular laboratory technician was blinded to patients' information and group allocation… DNA was extracted using the NZY Tissue gDNA Isolation Kit (NZYTECH) according to the manufacturer's instructions… Genotyping was carried out…using the iPLEX Gold technology”
Bias	9	Describe any efforts to address potential sources of bias	10, 11	“PD cases were diagnosed by experienced dentists… following standardized clinical procedures

				according to the 2018 AAP/EFP classification criteria…Immediately after PD diagnostics, a 2 mL peripheral blood sample was collected in EDTA-coated tubes from all enrolled participants (patients and controls), who were fully anonymized using a code by hospital personnel, so that the molecular laboratory tech-nician was blinded to patients' information and group allocation"
Study size	10	Explain how the study size was arrived at	10	"As this was an exploratory pilot study, no formal a priori sample size calculation was performed. All eligible women recruited during the study period who completed the study assessments and fulfilled the inclusion criteria were included."

Continued on next page

Quantitative variables	11	Explain how quantitative variables were handled in the analyses. If applicable, describe which groupings were chosen and why	11, 12	"A total of 10 logistic models were constructed, involving the five genetic variants under study: IL1RN rs4251961, TNFA rs1800629, IL1A rs1143634, IL1B rs16944, and IL1B rs1800587. For each gene, two models were considered, namely (i) the full models, with all three genotypic categories, and (ii) the 'collapsed models', in which the two possible genotypes with the minor allele were collapsed into one category, with the homozygote for the major allele being the alternative genotype (reference genotype)."
Statistical methods	12	(*a*) Describe all statistical methods, including those used to control for confounding	11, 12	"Statistical analysis of the questionnaire data…(using chi-square/Fisher's exact test or Student's t-test), was performed using R v.4.2.1. The chi-square test was used to evaluate SNPs within the healthy control group for potential deviations from Hardy-Weinberg equilibrium. Additionally, logistic regression models were used to assess the role of genetic predictors in explaining the presence or absence of PD. Firstly, the likelihood ratio test was applied to estimate the global effect of the predictors on PD. Secondly,

				whenever such an effect was significant, the Wald test was applied to determine whether the odds ratios for each predictor category were significant relative to a reference category. Adjustments to the significance of the results in each model were implemented using multiple correction methods, to control the family-wise error rate (FWER) and the false discovery rate (FDR) in view of the number of simultaneously tested hypotheses… All procedures for the logistic regression analysis were performed using IBM SPSS Statistics (version 29)."
		(*b*) Describe any methods used to examine subgroups and interactions	N/A	Not applicable
		(*c*) Explain how missing data were addressed	11	"…seven women were excluded due to missing data on the variables under study"
		(*d*) *Case-control study*—If applicable, explain how matching of cases and controls was addressed	N/A	Not applicable
		(*e*) Describe any sensitivity analyses	N/A	Not applicable
**Results**				
Participants	13*	(a) Report numbers of individuals at each stage of study—eg numbers potentially eligible, examined for eligibility, confirmed eligible, included in the study, completing follow-up, and analysed	11	"Among the 95 subjects, 33 women had PD. The remaining subjects, who did not meet the diagnostic criteria for PD, were eligible for use as healthy controls, but seven women were excluded due to missing data on the variables under

				study; thus, 55 women were enrolled as control subjects."
		(b) Give reasons for non-participation at each stage	11	"…seven women were excluded due to missing data on the variables under study"
		(c) Consider use of a flow diagram	N/A	Not applicable
Descriptive data	14*	(a) Give characteristics of study participants (eg demographic, clinical, social) and information on exposures and potential confounders	3, 4	"Table 1. Demographic and lifestyle characteristics of patients with PD and healthy controls."
		(b) Indicate number of participants with missing data for each variable of interest	11	"…seven women were excluded due to missing data on the variables under study"
		(c) *Cohort study*—Summarise follow-up time (eg, average and total amount)	N/A	Not applicable
Outcome data	15*	*Case-control study*—Report numbers in each exposure category, or summary measures of exposure	4, 5	"Table 2. Distributions of the three genotypic categories of selected SNPs (IL1A rs1800587, IL1B rs1143634, IL1B rs16944, IL1RN rs4251961, and TNFA rs1800629)."
Main results	16	(*a*) Give unadjusted estimates and, if applicable, confounder-adjusted estimates and their precision (eg, 95% confidence interval). Make clear which confounders were adjusted for and why they were included	5-7	"Table 3. Results of likelihood tests of global effects of genetic predictors: ordered p-values and their adjustments for the 10 logistic models"<br>"Table 4. Genotype distributions of IL1RN rs4251961 and TNF rs1800629 in patients with PD and controls, significances of the Wald test results and power calculations"
		(*b*) Report category boundaries when continuous variables were categorized	N/A	Not applicable

	(*c*) If relevant, consider translating estimates of relative risk into absolute risk for a meaningful time period	N/A	Not applicable

Continued on next page

Other analyses	17	Report other analyses done—eg analyses of subgroups and interactions, and sensitivity analyses	N/A	Not applicable
**Discussion**				
Key results	18	Summarise key results with reference to study objectives	8, 9	"in the present pilot study, we investigated the potential associations of IL1A rs1800587, IL1B rs1143634 and rs16944, TNFA rs1800629, and IL1RN rs4251961 with PD in a Portu-guese women cohort. The main findings of the present study were the associations between TNFA rs1800629 and IL1RN rs4251961 variants and PD. TNFA rs1800629 showed a moderately significant association with PD, while IL1RN rs4251961 revealed a strongly significant association with the disease in both the full and collapsed models, even after BH and Bonferroni corrections."
Limitations	19	Discuss limitations of the study, taking into account sources of potential bias or imprecision. Discuss both direction and magnitude of any potential bias	9, 10	"As expected, the main limitation of our pilot study is the sample size (N = 88), which may be insufficient for detecting moderate effect sizes, especially given the binary nature of the outcome. The power was further reduced by multiple testing adjustments, with Bonferroni, Benjamini-Hochberg, and Benjamini-Yekutieli corrections lowering the significance threshold,

				increasing the likelihood of Type II error. Additionally, the effect size, while notable (e.g., OR = 3.52 for the IL1RN/TC genotype), is not large enough to compensate for sample-size constraints, particularly if the distribution of subjects across genotype categories is unbalanced. The wide confidence intervals, especially for the IL1RN/CC genotype, further suggest instability in the estimates due to limited event counts. Another aspect to consider is the characteristics of the study population. Although participants were recruited during the postpartum period, pregnancy-associated hormonal and immunological changes are primarily linked to pregnancy-associated gingivitis, a transient and generally reversible condition, rather than PD [46]. Therefore, the present findings should be interpreted as genetic associations observed in this specific population. Further studies including women at other stages of life will be important to determine the generalizability of these findings."
Interpretation	20	Give a cautious overall interpretation of results considering objectives, limitations, multiplicity of analyses, results from similar studies, and other relevant evidence	10	Overall, this pilot study provides preliminary evidence supporting an

				association between IL1RN rs4251961 and PD within this Portuguese postpartum population. However, future studies with larger sample sizes are needed to confirm and validate this association in individuals of European ancestry. To increase the statistical power of future studies on this subject, collaborative research protocols involving dental clinic networks or governmental primary health care networks should be encouraged. Notwithstanding, the present study contributes to the understanding of the genetic background of PD susceptibility by highlighting IL1RN rs4251961 as a candidate variant for further investigation, while also emphasizing the importance of considering sex and ancestry in genetic association studies.
Generalisability	21	Discuss the generalisability (external validity) of the study results	10	"Although participants were recruited during the postpartum period, pregnancy-associated hormonal and immunological changes are primarily linked to pregnancy-associated gingivitis, a transient and generally reversible condition, rather than PD [46]. Therefore, the present findings

				should be interpreted as genetic associations observed in this specific population. Further studies including women at other stages of life will be important to determine the generalizability of these findings… future studies with larger sample sizes are needed to confirm and validate this association in individuals of European ancestry”
**Other information**				
Funding	22	Give the source of funding and the role of the funders for the present study and, if applicable, for the original study on which the present article is based	12	“This work was supported by FCT - Fundação para a Ciência e Tecnologia, I.P., in the scope of the project UID/04378/2025 (DOI identifier 10.54499/UID/04378/2025), and UID/PRR/04378/2025 (DOI identifier 10.54499/UID/PRR/04378/2025), of the Research Unit on Applied Molecular Biosciences - UCIBIO and the project LA/P/0140/2020 (DOI identifier 10.54499/LA/P/0140/2020) of the Associate Laboratory Institute for Health and Bioeconomy - i4HB. This work was also supported by FCT – Fundação para a Ciência e Tecnologia, I.P., through the Egas

			Moniz Center for Interdisciplinary Research (CiiEM) project UIDB/04585/2020 (DOI identifier 10.54499/UIDB/04585/2020)."

*Give information separately for cases and controls in case-control studies and, if applicable, for exposed and unexposed groups in cohort and cross-sectional studies.

Note: An Explanation and Elaboration article discusses each checklist item and gives methodological background and published examples of transparent reporting. The STROBE checklist is best used in conjunction with this article (freely available on the Web sites of PLoS Medicine at http://www.plosmedicine.org/, Annals of Internal Medicine at http://www.annals.org/, and Epidemiology at http://www.epidem.com/). Information on the STROBE Initiative is available at www.strobe-statement.org.
